# Conceptualizing Cancer Drugs as Classifiers

**DOI:** 10.1371/journal.pone.0106444

**Published:** 2014-09-23

**Authors:** Patrick Nathan Lawlor, Tomer Kalisky, Robert Rosner, Marsha Rich Rosner, Konrad Paul Kording

**Affiliations:** 1 Department of Physical Medicine and Rehabilitation, Rehabilitation Institute of Chicago, Northwestern University, Chicago, Illinois, United States of America; 2 Department of Engineering, Bar-Ilan University, Ramat Gan, Israel; 3 Department of Physics, University of Chicago, Chicago, Illinois, United States of America; 4 Ben May Department for Cancer Research, University of Chicago, Chicago, Illinois, United States of America; 5 Department of Physiology, Rehabilitation Institute of Chicago, Northwestern University, Chicago, Illinois, United States of America; Institute for Systems Biology, United States of America

## Abstract

Cancer and healthy cells have distinct distributions of molecular properties and thus respond differently to drugs. Cancer drugs ideally kill cancer cells while limiting harm to healthy cells. However, the inherent variance among cells in both cancer and healthy cell populations increases the difficulty of selective drug action. Here we formalize a classification framework based on the idea that an ideal cancer drug should maximally discriminate between cancer and healthy cells. More specifically, this discrimination should be performed on the basis of measurable cell markers. We divide the problem into three parts which we explore with examples. First, molecular markers should discriminate cancer cells from healthy cells at the single-cell level. Second, the effects of drugs should be statistically predicted by these molecular markers. Third, drugs should be optimized for classification performance. We find that expression levels of a handful of genes suffice to discriminate well between individual cells in cancer and healthy tissue. We also find that gene expression predicts the efficacy of some cancer drugs, suggesting that these cancer drugs act as suboptimal classifiers using gene profiles. Finally, we formulate a framework that defines an optimal drug, and predicts drug cocktails that may target cancer more accurately than the individual drugs alone. Conceptualizing cancer drugs as solving a discrimination problem in the high-dimensional space of molecular markers promises to inform the design of new cancer drugs and drug cocktails.

## Introduction

**Figure 1:**
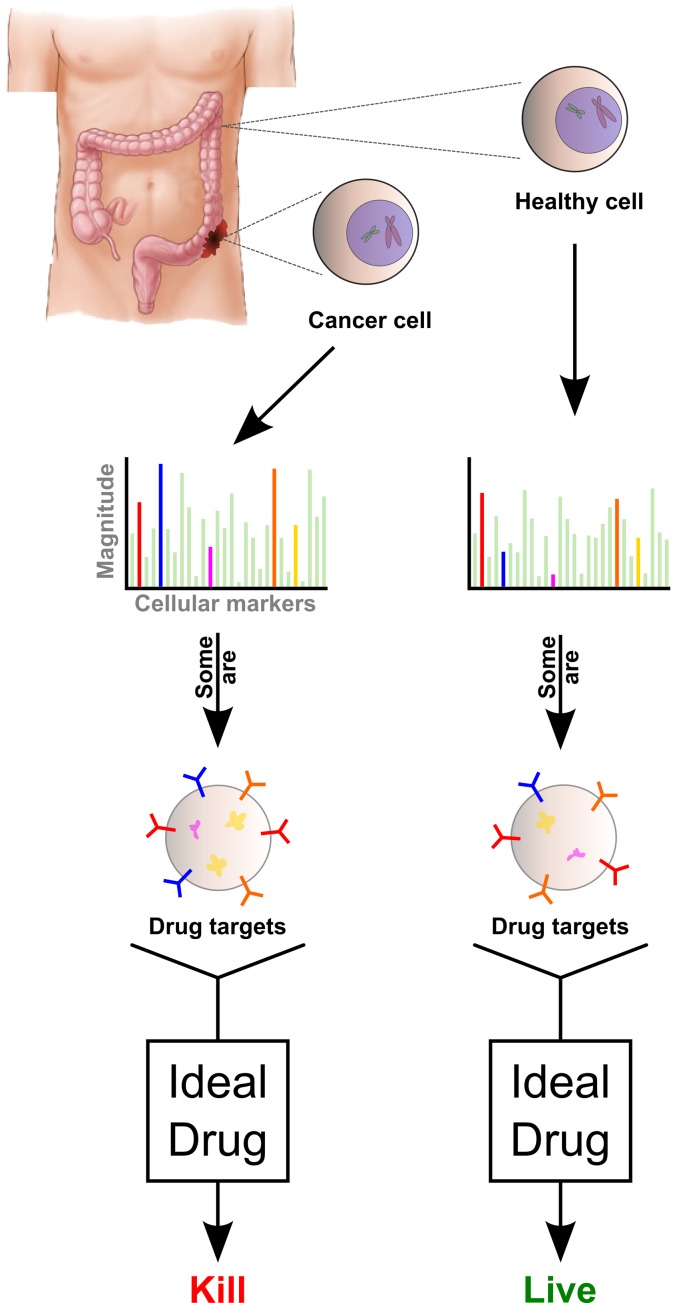
Cancer drugs solve a discrimination problem. Ideally, cancer drugs perform a computation on cells: kill (if cancerous) or no-kill (if healthy).

The central objective of treating cancer is to kill cancerous tissue while leaving healthy tissue intact. Effective cancer drugs must therefore distinguish between cancer cells and healthy cells. Additionally, optimal cancer treatment should also be robust to biological variability such as tumor and healthy cell heterogeneity [1]. Combining these ideas, we can frame the cancer problem in a way that balances the potential overlap of healthy and cancer cell properties with the need to kill aggressive cancer cell variants (Fig. 1). While the need to separate cancer from healthy cells underlies current cancer treatment, to our knowledge it has not been mathematically formalized. Developing a mathematical framework opens the possibility of translating insights from computational science into new approaches for cancer treatment.

**Figure 2:**
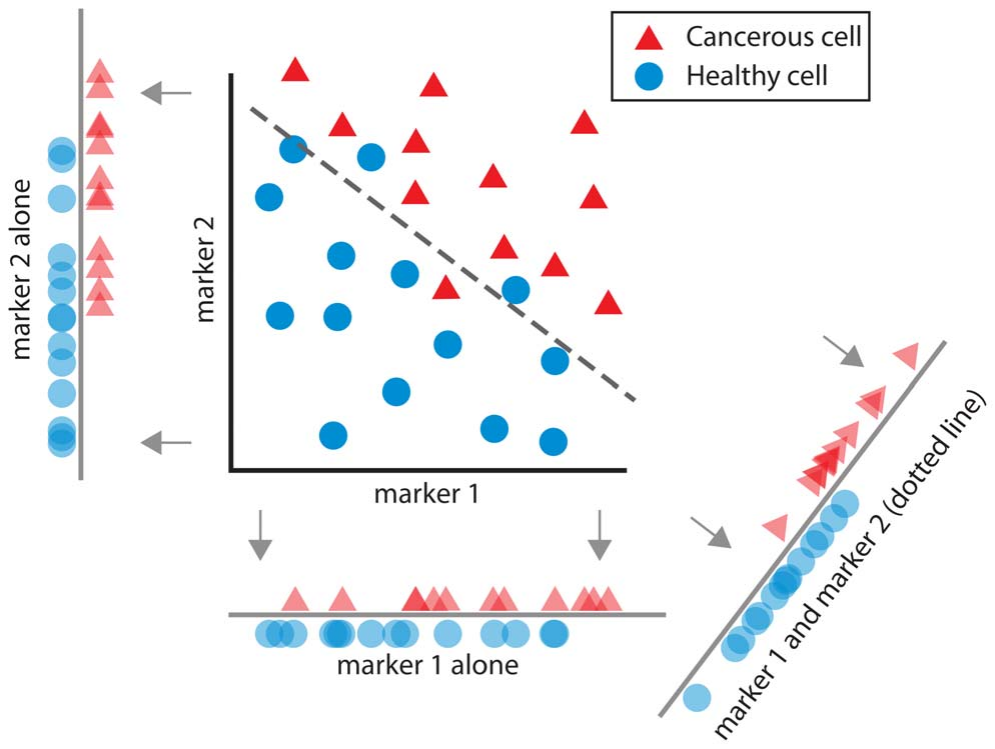
The idea of a classifier. This illustrates how one can combine information from two cellular markers to construct a classifier that separates the two populations (cancerous and healthy cells) better than either marker alone.

Cancer drugs should thus be conceived of as performing a computation on cells. For example, for toxic drugs, cellular targets lead to a single outcome (kill or do not kill) during treatment. Mathematically, we can say that the effect of a drug is a mapping from a set of properties (targets of the cell) onto a stochastic, binary outcome (the cell lives or dies) - this is exactly the definition of a classifier in the fields of statistics and machine learning [2]. In that sense, any cancer drug is actually a classifier (Fig. 2). However, the application of the word “classifier” to this selective killing is not just semantic. Instead it relates to a formal mathematical approach and toolbox derived from machine learning, which can contribute to drug development.

Many computer algorithms have been developed to solve classification problems, and a rich literature exists in the fields of statistics and machine learning regarding effective methods for classification [2]. These computational fields offer a broad range of approaches including quantitative performance metrics, efficient algorithms for large datasets, and methods for improving classifiers. For example, much research has addressed how to combine weak classifiers in order to build better classifiers, suggesting that these methods can be adapted to drug combination (Fig. 2). Machine learning, in defining the search for classifiers as optimizers, offers a clean way of describing a goal-directed search. This paper is meant to clarify the equivalency of the search for classifiers and the search for cancer drugs.

Data from newly developed Omics approaches enable the application of classifier theory to drug optimization. Microarray and sequencing technology, for example, allow us to simultaneously collect information about thousands of cellular *markers* – measurements that characterize the state or phenotype of the cell such as gene expression. Some of these markers should distinguish cancer cells from healthy cells, aiding in accurate classification and cancer targeting. Importantly, however, not all markers are molecular drug targets. Molecular *targets* are molecules that cancer drugs actually use to alter cells. But buried in the thousands of measurable markers is a subset of markers that *reflect or correlate with* the molecular targets of drugs. For example, expression of genes that are downstream of a drug target may correlate well with that drug's efficacy. Emerging biotechnology allows us to measure these cellular markers and analyze them by statistical tools like machine learning to more fully understand cancer.

Although cancer drugs have not been formally characterized as classifiers, machine learning has been extensively applied to many aspects of cancer biology. One group has estimated breast cancer outcome by using machine learning to create a 70 gene prediction algorithm [3] while we and others have used machine learning, in combination with discrete signaling pathways, to predict metastasis-free survival [4]. Others have attempted to distinguish between different types of cancer using many types of algorithms including Support Vector Machines (SVM) [5], [6], Principal Component Analysis [7], [8] and Artificial Neural Networks [9]. Yet others predict chemosensitivity on the basis of gene expression [10], [11] and signaling networks [12]. However, while all these approaches have made impressive strides and are useful in clinical practice, these ideas have not been combined to produce a principles-based approach to cancer drug design.

Here we propose a framework for designing cancer treatments that extends existing ideas using the classifier conceptualization. We first outline the approach, and then, as a practical example, carry out an analysis of experimental data to show how this can be done in principle. The overall approach is summarized below:

### A Framework for Treating Cancer

Formalizing cancer drugs as classifiers should inform how we treat cancer. There are three essential parts of this approach that we first summarize at a high level, and then demonstrate using currently available experimental data: 1) Specifically define the objective to be achieved by classification - namely, which cells to kill, which cells to leave, and how to tell the difference between the two; 2) Understand the treatment tools at our disposal; and 3) Optimally use these treatment tools to accomplish the defined classification objective. We have summarized definitions and assumptions related to this framework in Table 1.

**Table 1:** 

Definitions	
Molecular marker	A measurable cellular quantity. E.g., gene expression, protein expression, epigenetic markers.
Molecular target	A cellular entity allowing or causing drug action on the cell.
Optimal drug	Hypothetical drug that would perfectly discriminate between cancer and healthy cells. I.e., would kill all cancer cells while leaving all healthy cells alone. Used as a guide for designing treatment.
**Assumptions**	
Linearity of drug combinations	If two drugs are administered together, their respective mechanisms will be roughly unchanged by the presence of the other drug. Allows prediction of drug combination effects from individual drug effects.
**Simplifications**	
Single cells vs cell lines	Ideally, we would approach cancer treatment from single-cell perspective. We use single-cell data where possible, and use cell lines as substitutes as necessary for demonstration purposes.

### Defining the objective (Part I)

The first step is to discriminate between cancerous and healthy cells. Because this is the goal of cancer treatment, it is important to concretely specify this goal. To do this, we use the mathematics of classification algorithms in conjunction with measurements of cell markers. The classifier answers the following questions: how much do cancer cells differ from healthy cells, and which biological markers can distinguish them? It asks this question while explicitly considering the heterogeneity of both populations. The markers could include gene expression, surface proteins, etc. Because distinguishing between cancer and healthy cells requires taking into account the heterogeneity in each population, we have focused on markers for single cells rather than cell populations where possible.

Classifying algorithms in this context are designed to give the maximal separation of cancer cells from healthy cells in terms of these distinguishing markers. As such, we can say that the results of classification describe what a hypothetical “optimal” drug acting upon these markers could achieve. Part I of the Results demonstrates how one could define this optimality objective using gene expression in single cells and explores how many markers and cells are required to achieve this goal.

### Understanding the treatment tools at our disposal (Part II)

The next step is to understand how the available treatment tools allow us to utilize the distinguishing markers of cancer cells. We should strive to approximate the optimal drug by designing new drugs or combining existing drugs. Here we focus on the second.

Drugs should ideally target distinguishing properties of cancer, but most drugs used in the clinic do not do this perfectly. Furthermore, their mechanisms of action differ. Thus, it may be possible to predict how existing drugs should be combined to produce more desirable results. Actually making this prediction would hinge on relating the drug actions to the distinguishing properties of cancer. For example, if cancer cells differ from healthy cells primarily via three distinct markers, three drugs *known* to separately utilize each single marker as a classifier could theoretically be combined. Part II of Results demonstrates this idea by exploring the relation between drug action and molecular properties (gene expression).

### Optimally using treatment tools (Part III)

The last step is to use the mathematics of classification to ask how our available tools (e.g. drugs) allow us to accomplish our objective. From the first part, we know how to distinguish cancer cells from healthy cells. From the second, we know how our drugs relate to molecular markers. Now we can use these two ideas to optimize treatment by matching the classifying abilities of our drugs to the specified classification objective.

Here we will again focus on optimizing drug combinations. One approach would be to predict drug combinations that discriminate between cancer and healthy cells better than either drug alone. Part III of Results demonstrates one approach that, under ideal conditions, might achieve this goal.

One limitation of this study is that the data currently available for such analyses are limited and not ideal. Therefore, in order to illustrate the three parts of the classification framework using data from real biological samples, we have substituted or redefined cell phenotypes when the desired cell data is lacking.

## Results

### Part I: Defining the classification objective with molecular measurements

If cancer drugs act as classifiers that use measurable markers as input, we can use standard classification algorithms to explore the possibility of solving the cancer versus healthy cell classification problem. It is theoretically possible that there is an optimal drug (or drug combination) that achieves this goal in practice. This would imply that such a drug would kill cancer cells while leaving healthy cells alone to the greatest extent possible. We will use this notion of an optimal drug as a guide to analyzing treatment. In practice, actual drugs or drug combinations should be chosen to resemble the optimal drug. We recognize that every tumor is different, but our goal here is to illustrate an approach for distinguishing cancerous from non-cancerous tissue in one context. This approach can also be applied to other cancer types.

To determine if it is theoretically possible to solve this problem, we need a dataset of cells with both known cancer state and measured markers. We used single-cell transcriptional data derived from the colon [13]. This dataset included both a limited number of markers (45 genes), and a limited number of colon tissue subtypes and cells (<200 cells). Thus, we focused on distinguishing between healthy and cancerous cells of one tissue subtype: stem-like cells. Because these cells are so similar – as pointed out in the original publication – this choice served to make the classification problem more challenging. This dataset thus allowed us to test the power of the classification approach.

Can this classification problem be solved? In other words, can the single-cell transcription data predict cell state (cancerous or not)? To answer this question we used a standard classification algorithm, the regularized GLM [14], [15]. Testing how well such classifying algorithms work allows us to give an upper bound on how well an actual drug could work if it used gene expression alone.

**Figure 3:**
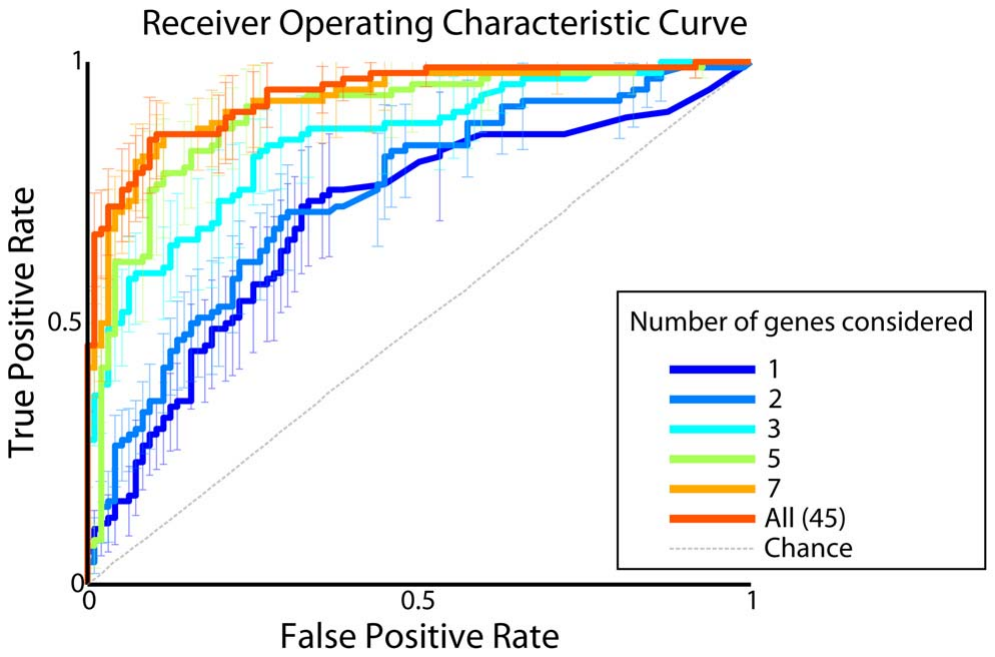
Discriminability of healthy versus cancer cells as a function of the number of genes considered. When measuring accuracy of cell classification as cancerous or healthy, one should consider both types of errors: false positives and false negatives (or more conventionally, true positives). This is illustrated by the Receiver Operating Characteristic (ROC) Curve. Lines indicate mean values, and error bars indicate bootstrapped 95% confidence intervals. Accuracy was measured using cross-validation; and chance value was determined using shuffle control.

First, we wanted to measure the potential accuracy of this classification. In classification there are different kinds of errors that one can make. For example, it is easy to produce a drug that kills all cancer cells but also kills all healthy cells. This drug would have 100% true positives (killed cancer cells), but also 100% false positives (killed healthy cells). Thus, to fully characterize a classification strategy we should analyze the relation of the two types of errors. The standard measure of classification accuracy is the receiver operating characteristic (ROC) plot. In this plot the proportion of true positives is plotted as a function of the proportion of false positives (Fig. 3) to quantify both sensitivity and specificity. The area under this curve (AUC) gives an overall measure of classification performance with a maximum value of 1 for a perfect test. The AUC for our classification algorithm was ∼0.9, indicating that healthy and cancerous cells can be well classified. Therefore it is theoretically possible to solve this particular cancer versus healthy classification problem for single cells with high accuracy using expression levels for just a small set (∼45) of genes.

**Figure 4:**
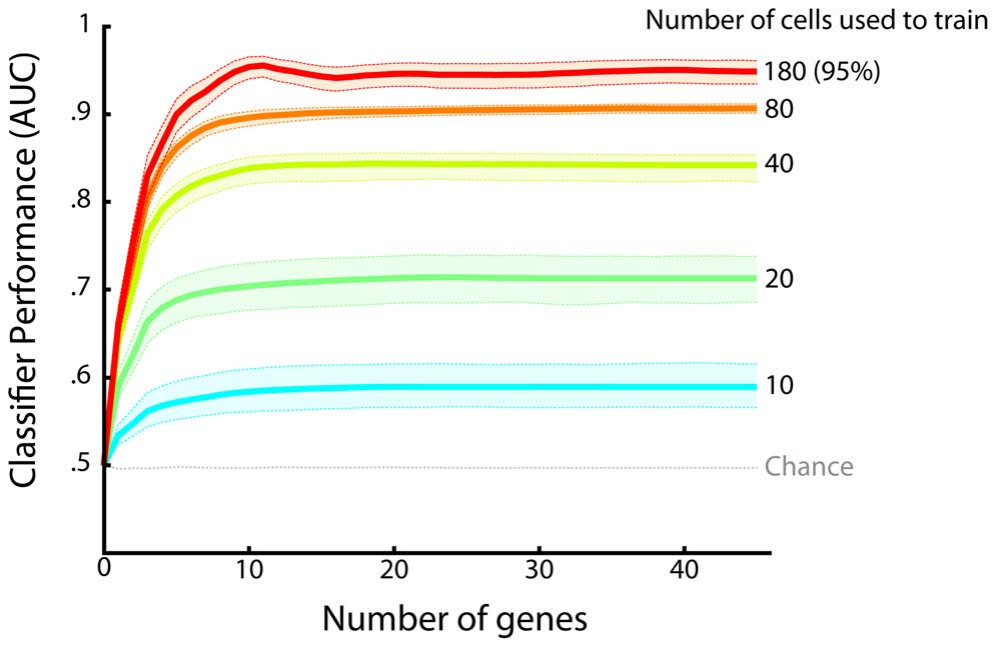
Discriminability of healthy versus cancer cells as a function of the number of cells and genes measured. Classification performance was measured as area under the curve (AUC) of the ROC curve. A perfect classifier would achieve an AUC of 1, whereas a random classifier would achieve an AUC of.5. Each colored line represents a different number of cells used to train the classifier, showing that performance improves as more cells are used. Lines indicate mean values, and shaded areas indicate bootstrapped 95% confidence intervals. Accuracy was measured using cross-validation; and chance value was determined using shuffle control.

Next we asked *how many* cellular markers an optimal drug would need to classify cells accurately. In other words, what is the minimum number of markers an optimal drug must consider to tell the difference between healthy and cancer cells? We found that a relatively small number of genes - approximately the best 10 (ranked by magnitude of fit parameter) - sufficed to classify a cell as cancerous or healthy with high accuracy (Figs. 3, 4). The predictive power of the classifier saturated soon after the best 10 genes were included. Thus, only a small number of cellular markers provide the majority of the information used to classify a cell as cancerous or healthy.

The ability to measure gene expression from single cells raises the question of whether it is more important to measure more cells or more genes. To answer this, we quantified the relative importance of increasing the number of measured cells versus the number of measured genes per cell. We trained the classifier with numbers of cells ranging from two total (one healthy, one tumor cell) to 180 total cells (95% of cells). As above, we measured the classifier's performance, except that we did so for each training scenario. We again found that the performance saturated after a small number of genes for each training scenario. Importantly, we also found that performance continued to improve with increasing numbers of training cells until approximately 80 were used (Fig. 4). Thus, measurements from at least tens of cells are required to account for variance in a simplified population of tumor and healthy cells.

In this section, we have demonstrated how to carry out an analysis that would define what optimal treatment – the “ideal drug” – could accomplish. With one experimental dataset we showed that it is possible to accurately solve the cancer classification problem. This type of analysis could also identify the markers that distinguish cancer from healthy cells. For the limited dataset that we chose, it appeared that solving the classification problem was possible with approximately 10 markers and 80 cells. However, these numbers will vary by tumor cell population as well as the particular markers analyzed.

### Part II: Classification by real cancer drugs

In the first part of Results we have explored the possibility of an optimal cancer drug that distinguishes between healthy and cancer cells. In this second part of the study, we will demonstrate *whether* and *how* the tools at our disposal (in this example, actual drugs) can classify cells. This will help us understand how to use actual drugs to approach the performance of the optimal drug.

Specifically, in the following example, we ask how cancer drugs *actually* relate to molecular markers. To answer this question, we would ideally use single cells. This is difficult with single cells, however, because treatment and marker measurement both potentially destroy the cell. A possible solution is to enable limited replication of single cells in order to analyze marker status for one set of daughter cells while simultaneously determining drug efficacy using other daughter cells. However, even these two sets will exhibit heterogeneity with time. Since gene expression data for such populations that have been treated with drugs are not presently available, the closest substitute is established cell lines that have a clonal origin and are largely genetically homogeneous. Therefore we used cell lines as stand-ins for single cells to ask how drugs relate to molecular markers.

Because our previous results suggest that we need tens of cells to solve the classification problem, we chose to analyze a type of tissue with many established cell lines. We therefore used 45 luminal and basal-like breast cancer cell lines characterized by Gray and colleagues [12]. They measured approximately 19,000 genes in these breast cancer lines using microarrays, as well as the chemotherapeutic responses of those lines to each of 74 drugs. These lines are a good representation of the range of cell phenotypes found in breast cancer and thus represent more variance than an actual tumor. Nonetheless, these cell lines allowed us to ask how cell markers related to drug sensitivity.

We used an algorithm to predict if cancer drugs will kill cells of a specific cell line on the basis of its markers. In particular, we estimated the drug sensitivities of these cell lines. Indeed, some aspects of the drug responses were predictable. For example, we predicted the chemotherapeutic response to the drug Lapatinib, a tyrosine kinase inhibitor that blocks signaling by both the EGF receptor and HER2/neu. We obtained an R^2^ value of ∼0.5. However, the low R^2^ value shows that our prediction of drug behavior was not strong. This could be due to the small size of the dataset, but may also imply that not all relevant markers were measured, or alternatively that the relationship is nonlinear and not captured by linear machine learning methods. While molecular markers such as gene expression do not capture all variability, they do appear to play a role in predicting the drug response. Therefore, cellular markers predict some degree of drug behavior when treated as inputs to a classifier.

**Figure 5:**
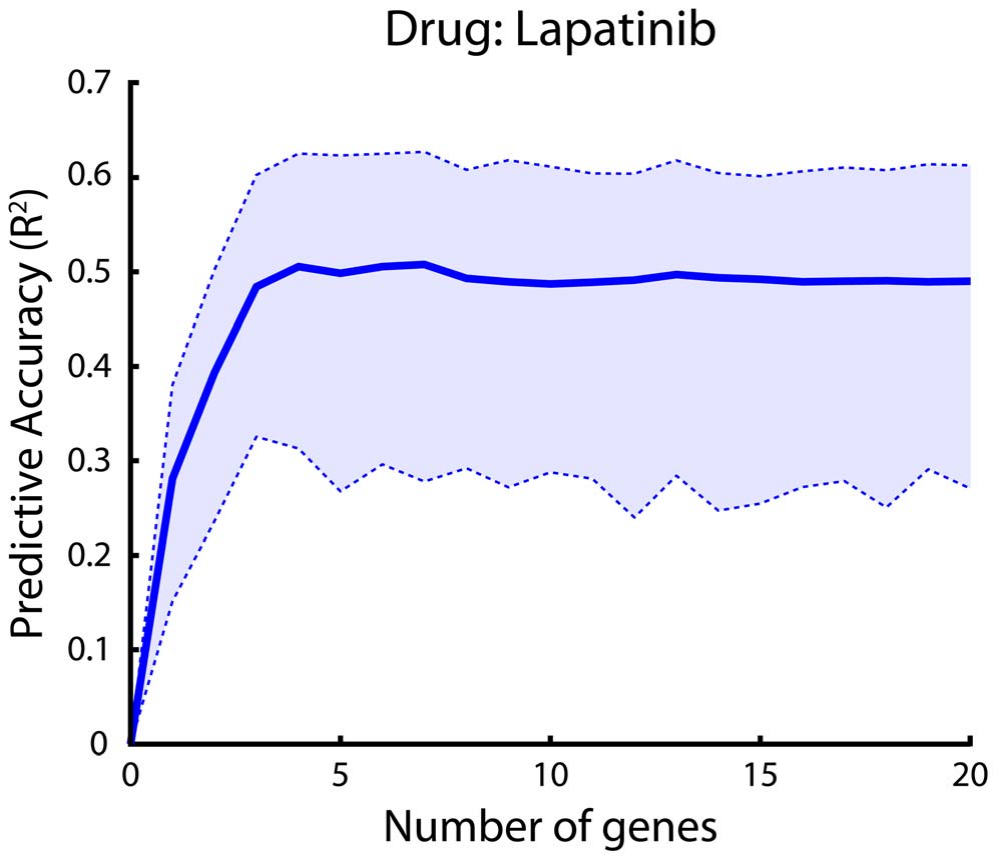
Drug response prediction as a function of the number of genes considered. Drug response was predicted using molecular markers (gene expression). Accuracy of chemosensitivity prediction, measured as R^2^, which represents the amount of variation explained. Shaded areas indicate bootstrapped 95% confidence intervals. Accuracy was measured using cross-validation.

We also wanted to know how many genes suffice to predict actual drug behavior. To do this, we measured the drug's behavior as a function of the number of genes. Lapatinib needed only a small number of genes (∼5) to reach its peak accuracy (Fig. 5). Thus, a small number of cellular markers predict whether or not actual drugs kill a cell. This is important because it shows that we do not necessarily need to consider many thousands of genes when designing therapies.

**Figure 6:**
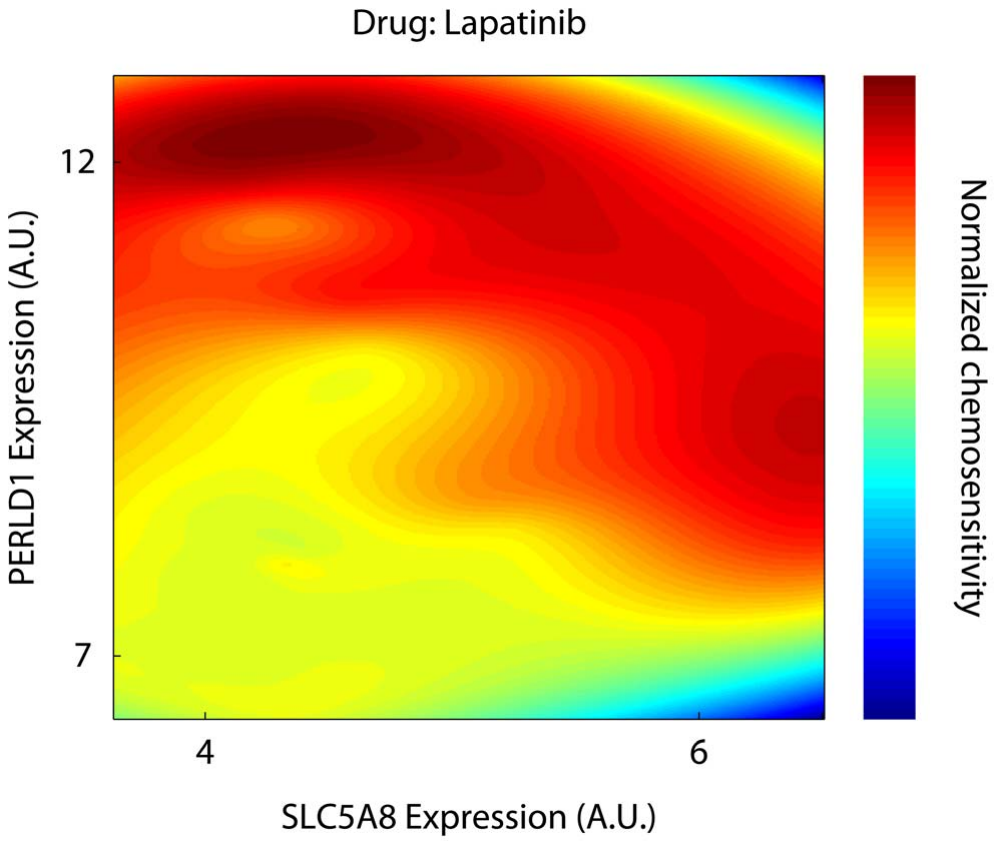
Drug sensitivity heatmap as a function of the two most important genes. Drug activity is to some extent predictable using molecular markers.

If drugs act as classifiers using a small number of properties, then a drug can be characterized by plotting its effect as a function of the properties. We thus selected the two genes (SLC5A8 and PERLD1) that were jointly best at predicting Lapatinib's effect across cell lines. Using simple grid interpolation and extrapolation routines we plotted the drug's effect as a heatmap (Fig. 6). This type of visualization shows that even two genes can capture the complex behavior of a cancer drug.

We analyze drug activity as it relates to molecular markers (i.e., phenotypic characteristics of a cell), but such markers are not necessarily molecular *targets* (i.e., cell components such as proteins whose functions are altered by a drug). Ideally, experimental data would also include measurements of markers more likely to actually constitute drug targets such as the tyrosine kinase receptors that are targeted by Lapatinib. Although the gene transcripts we analyze are not the direct targets of the classifying drug, they still predict drug response and are therefore useful. Thus, this approach could be used with *any* type of molecular marker or targets.

In this section, we have demonstrated how to understand the behavior of actual drugs within our framework. In our example, we related drug efficacy to molecular markers which, in theory, allows one to ask how actual drugs relate to the distinguishing markers of cancer cells. This will be important for the next section in which we ask how to optimally combine drugs. In this dataset, however, we find that the measured markers suboptimally predict the drug response. In the future, data collected from many more cell populations and a variety of marker types will be needed to make clinically plausible predictions.

### Part III: Optimizing cancer treatment

In the previous two sections we have shown how an optimal cancer drug could classify cells as cancerous or not, and that cellular markers predict to some extent how an actual drug behaves. In this section, we combine these two ideas to outline a possible approach to optimizing cancer treatment. We look for *pairs of drugs* that classify cancer cells better when combined than either drug alone.

We demonstrate this approach conceptually using gene expression data and drug sensitivity measurements from the same panel of breast cancer cell lines [12]. Unfortunately, we were not able to obtain similar data for noncancerous cell lines. Therefore we made two adjustments to show that these types of analyses are possible in principle: 1) we again treated each clonal cell line as a stand-in for a single cell; and 2) we used the ability to discriminate between two subclasses of tissue as the classification objective rather than distinguishing healthy from cancer cells. In particular, we used the basal-like and luminal subtypes of breast cancer, which *roughly* correspond to aggressive (more metastatic) and less aggressive (less metastatic) cancer. We are aware that the real situation is more complex than this simplification; however this distinction is ultimately arbitrary and simply serves to demonstrate our approach using a real phenotypic difference. Given this definition and these data, we asked if it was possible to find two drugs that discriminate between the luminal and basal-like breast cancer subtypes better than either drug alone.

Real drugs do not perfectly discriminate between healthy and cancerous cells. However, we can use machine learning to describe how to combine drugs in order to better approximate the optimal drug. This is inspired by the well-known approach in machine learning called boosting [16], in which additional features are added to a classifier to enable progressively better performance. More specifically, given the classification objective (optimal drug) from Part I and drugs' actual behavior from Part II, we can determine which pair of drugs best approximate the optimal drug. To do this, we again use the GLM framework to frame the desired treatment as a combination of drugs. We then iterate through possible two-drug combinations to determine which pair performs best. This idea allows us to determine the best drug combination for classification.

**Figure 7:**
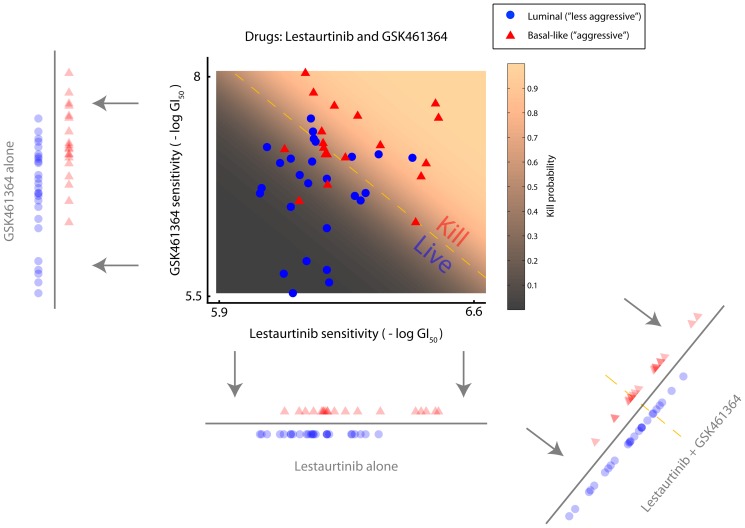
Cancer treatment optimization. Better discrimination between cell populations is achieved by including an additional drug. The classification threshold line shown, in reality, represents a gradient related to “probability of cell death” which is indicated by shading. See text for full description.

**Figure 8:**
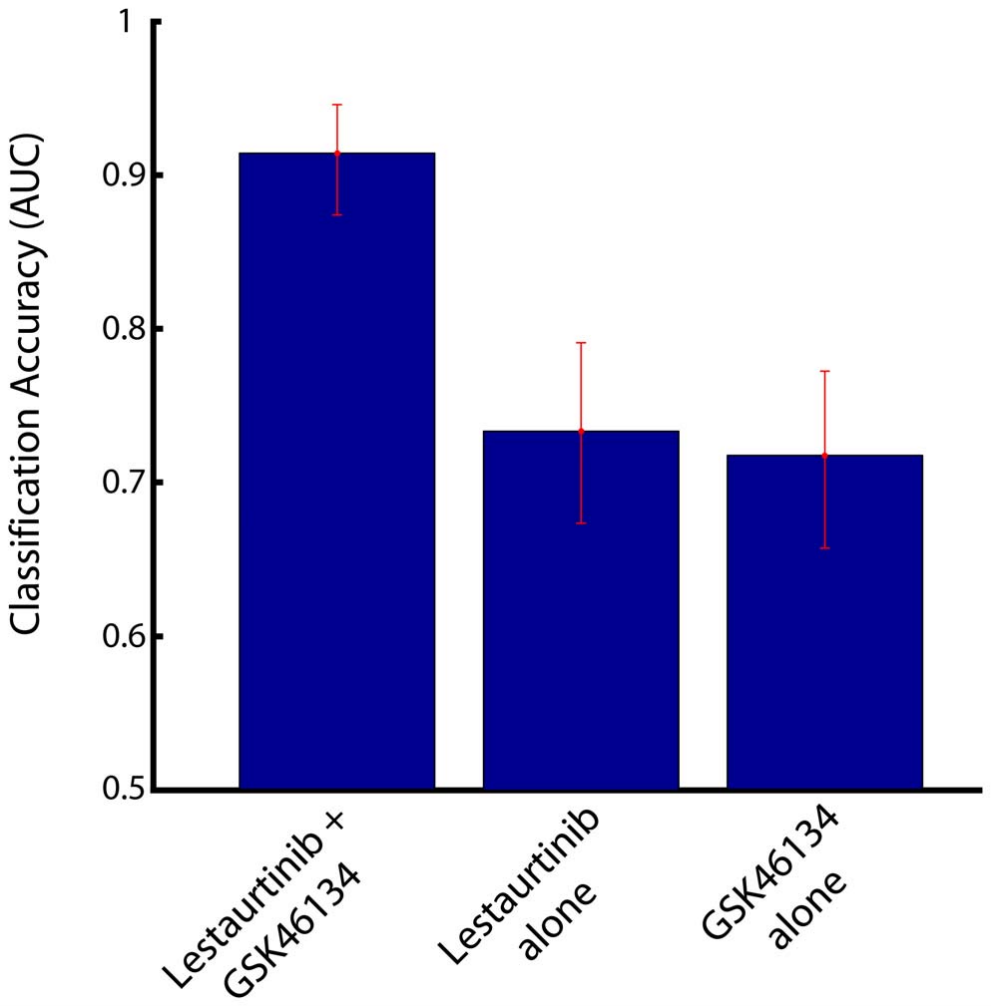
Classification accuracy showing the improvements achieved by using an additional drug. Accuracy (AUC) achieved by both drugs together is better than either drug alone.

We found several drug combinations that approximated the optimal drug. One particular combination included the drugs Lestaurtinib and GSK461364 (Fig. 7; compare Fig. 2). These drugs together provide a better classification than either drug alone (Fig. 8). Thus our method provides a mechanism for choosing additional drugs in a way that should allow us to target cancer cells more effectively. These results assume that the drugs act both independently and prior to an adaptive response to the treatment. Other strategies for addressing this issue are presented in the discussion.

In this section we have demonstrated how to optimize treatment using the classification framework. We emphasize again that we are using suboptimal biological data as examples to clarify the nature of our approach, not to produce clinically relevant predictions. Larger and more exhaustive datasets will be needed to make this possible. Furthermore, we have made simplifications unlikely to generalize to clinical practice. Thus, these results should not be taken as a clinical recommendation. Nevertheless, this analysis shows that using the classification framework to optimize treatment takes into account the inherent variation in phenotypes and could influence choice of a treatment to discriminate between cancerous and healthy cells.

## Discussion

### The approach for treating cancer

In this study, we have argued that employing cancer drugs as classifiers provides a conceptual framework for devising optimal treatment strategies for cancer. Optimal drugs use molecular targets to kill cancer cells while minimizing harm to healthy cells. We considered this problem as one that could be addressed with tools from machine learning and demonstrate how this could inform a strategy for treating cancer. We demonstrate that one class of molecular markers, gene expression, was sufficient to solve this optimization problem quite well using the data sets examined. We also showed how to incorporate intrinsic cell variation into the analysis and recognize actual drugs as suboptimal classifiers. Finally, we suggested ways of using the classification framework to derive drug development strategies that perform as closely as possible to an optimal drug.

Optimizing cancer treatment by combining drugs according to classification principles is relatively straightforward if combined drugs do not affect one another. For example, it may be that a second drug does not significantly interfere with the molecular mechanism of the first, and *vice versa* when administered simultaneously. If the effects of the individual drugs are additive, the ability of a particular drug to classify cancer cells would not be affected by another drug. Thus the compound classifier – the drug combination – would classify cancer cells more accurately than either drug alone. It is also possible that weak nonlinear interactions between drugs could still yield a superior compound classifier than either drug alone. Assuming linearity places an *upper bound* on how well drug combinations could work.

What if we find a second drug that should improve classification but is not additive with the first drug? This nonlinear dependency is very likely to be important. One solution would be to iteratively use the next-most-optimal drug until we find one that does not significantly interact with the first drug. Thus, choosing the best additional drug for a combination may require theoretical and empirical considerations. This method cannot guarantee that a given combination of drugs will work but instead proposes a more efficient way to select drug cocktails for testing. Another way to address this problem is to consider sequential as opposed to simultaneous drug treatment. Initial molecular marker profiles can be used to identify drug A as the optimal single drug. After exposure to drug A, the marker profiles of these cells will likely change as a result of the treatment. One could then utilize the altered marker profiles in cells that have been treated with drug A to identify the next optimal drug, B. In this regimen, the two drug treatments are by definition independent. Interestingly, a recent study by Yaffe and colleagues showed that the killing of aggressive, triple-negative breast cancer cells was more effective when two drugs of interest were applied sequentially rather than in combination [17]. By approximating the optimal treatment strategy using sequential drug administration, one may avoid the potential complications of drug interactions.

While we have defined the objective of a cancer drug as killing cancer cells, we realize that the goal of anticancer treatment may be more nuanced. In reality, drugs will kill cells with a certain probability (see Fig. 7). The goals of treatment may be controlling tumors rather than killing *per se*. For example, one may aim to prevent metastasis, or to slow or stop tumor growth. Although the treatment goal will ultimately depend on the clinical scenario, effective treatment strategies hinge on achieving a differential response between healthy and cancerous cells. Classification principles could aid in suggesting possible drug combinations to achieve other desired differential outcomes in addition to cell death.

### Limitations

This study also highlights the limitations of experimental data currently available. First and foremost, data collected from many more cell populations and a greater variety of molecular markers will be needed to make clinically relevant predictions. In this study, in order to examine both single cell as well as treatment data, we analyze data from two different organs. The first section uses a dataset from intestinal tissues whereas the second and third sections use a dataset from breast cell lines. While these choices prevented direct comparison of molecular markers across sections of this study, they were sufficient to demonstrate the application of the conceptual framework we propose. It should be noted that luminal breast and colon cancers are generally considered more treatable than other types (such as pancreatic cancer), raising the possibility that our proposed scheme may be more effective in some contexts than others. However, some properties of cancer are likely to be true regardless of the particular organ system [18]. If cancer drugs behave as classifiers for colon cancers, they will likely behave as classifiers in other organs. Analyzing cells originating from different tissues is therefore not likely to affect the principle of this study.

Another limitation of this study is that we measured markers in different tissue contexts. Unlike the Quake dataset (Part I), the Gray dataset (Parts II and III) measured expression of cultured cell lines rather than expression levels in primary single cells. Additionally, because this dataset did not include healthy cells, we had to demonstrate our approach by targeting one subtype of breast cancer versus another, rather than cancerous versus healthy cells. More rigorous analyses based upon treatment of single-cell or minimally amplified clones as well as relevant classification analyses will have to wait for more extensive experimental data to become available.

An important question that this study highlights is whether experiments with tumor cell *populations* capture as much relevant information as experiments with *individual cells*. Cell line gene expression levels reflect a population average rather than the expression of individual cells. It is possible that small numbers of drug-resistant cells with distinct marker profiles may not be detectable when the whole tumor cell population is analyzed together. For example, small populations of cells within a tumor have been shown to drive the evolution and drug resistance of some types of cancer [1], [19]–[21]. But although single cell data is preferable, it presents challenges of its own. Even genetically identical single cells can differ phenotypically in a number of ways including epigenetic state, protein and gene expression and morphologic state, and these states can change with time [22]–[24]. Thus, nongenetic heterogeneity across single cells may complicate classification analyses. These factors underscore the need to measure a diverse set of markers from single cells in order to target cancer effectively.

### Relation to previous work

Our statistical analyses have some similarity to other cancer studies that used statistical approaches in conjunction with large datasets. The Friend group, for example, used machine learning to predict prognosis and phenotypes in breast cancer [3]. This resembles our strategy in that they use gene expression to make predictions about cancer. Other groups have used machine learning to distinguish between sub-types of cancer [5]. They typically combine microarray data with such algorithms [6], [25] to distinguish between types of cancer. This resembles our strategy to *optimally* discriminate between cancer sub-types. The Golub group used machine learning to classify cell lines as belonging to a drug-resistant or drug-sensitive class based upon gene expression [10] whereas the Gray group related certain signaling networks to drug response [12]. More recently, a large consortium predicted cancer drug sensitivity using gene expression [11]. These studies have a similar goal of modeling drug responses. The Nowak group outlined a way to design drug cocktails that are robust to genetic mutation [26], while the Lauffenburger group designed them to minimize heterogeneity [27]. These studies likewise used cell populations as a source of phenotypic data; by contrast, we emphasize the need to ultimately consider single cells. These studies also stop short of using quantitative approaches to suggest a framework in which *cancer drugs themselves are classifiers*.

In contrast to these lines of research, we highlight a process by which potential targets for drug optimization can be identified *by defining discriminability as the drug's objective*. We use this property as a rationale for drug combinations that should optimize drug efficacy. We also demonstrate that one can design optimal drug classifiers using single-cell data that reflects normal and tumor cell heterogeneity. Conceptualizing drugs as classifiers is not only meaningful for cancer treatments. Any drug that should produce a binary outcome could be modeled using the same framework. This framework should generalize to drugs that are supposed to attack pathogens such as malaria, or undesirable cells, such as those responsible for asthma attacks [28].

### Moving cancer treatment forward

Thinking of drugs as classifiers should enable new advances in the treatment of cancer. For example, it may ultimately be possible to design extremely specific drugs based on targeting particular markers of interest. In essence, one would design a drug that requires cells to meet multiple conditions before being destroyed [29]. For example, these conditions could simply correspond to the expression levels of the ten genes most predictive of cancer. Many cells would likely meet one or two of the conditions but exceedingly few would meet all of them, ensuring that the drug only destroyed the cells it was designed to destroy. Although no such drug has yet been created, designing or screening for drugs that effectively perform classification could yield treatments that far surpass the abilities of current drugs and with fewer side effects.

This framework for cancer therapy may also extend to more complicated treatment scenarios. Although we have analyzed static molecular markers, real-world cells changes dynamically over time and in response to treatment [30]. It may be possible in the future to design treatment *schedules* based upon classification principles, better allowing us to combat or prevent drug resistance, for example. Furthermore, advances in experimental technologies will make it possible to measure a wider array of molecular markers, especially from single cells, enabling a better understanding of drug treatment. Our work thus represents only a first step and an extensible platform for thinking about cancer. Ultimately, collecting and analyzing the right types of datasets will continue to pose a challenge moving forward, and we believe that this study represents a useful way of framing both the questions and challenges associated with these fields.

## Methods

In this paper, we frame cancer drugs as solving a classification problem. As such, we approach this problem with the tools of machine learning, a field that routinely addresses these types of questions. Here we apply the Generalized Linear Model (GLM) framework, a machine learning algorithm, to explore the behavior of cancer using two gene expression data sets.

### Data

We used one dataset to explore how cancerous and normal tissue differ [13]. It was collected by Dalerba *et al* and provided by the Quake lab. They measured gene expression by performing PCR on single cells isolated from healthy intestine, primary intestinal tumor, and a xenograft derived from primary intestinal tumor tissue. Because their technology allowed them to measure the expression of only a relatively small number of genes (∼50), they chose these genes related to their goal of distinguishing between three subtypes of intestinal tissue (stem cells, and two differentiated cell types). More specifically, they chose the markers on the basis of a) reported association with tissue subtypes in the literature and b) an iterative method that distinguished between these subtypes. This means that the genes were optimized to distinguish stem-cells from non-stem cells, and not cancer from non-cancer cells, making our machine learning problem more difficult.

Because differences between healthy and cancerous tissues may be subtle, we sought to explore differences between the most similar groups of cells we could find in the data set. The original study used principal component analysis to identify sub-populations within the healthy, primary tumor, and xenograft tissues. We chose one particular sub-population that existed in both healthy and primary tumor tissues: stem-like cells. We then tested whether we could use a GLM to reliably identify which cells belonged to the healthy tissue and which belonged to tumor tissue, and how many markers were needed to do so.

We used another dataset to ask if actual cancer drugs act as classifiers and to determine how to optimize cancer treatment. This was collected by Gray, et al. for [12]. They measured the expression of approximately 19,000 genes in breast cancer tissues using microarrays, and the chemotherapeutic responses of those tissues. The breast cancer tissue came from a panel of 45 breast cancer lines. After gene expression of each cell line was measured, each line was treated with one of 74 drugs. They defined the sensitivity of a cell line to a given drug as the concentration of drug at which 50% of cell growth was inhibited (more specifically, they took the negative log of this number). We thus have a dataset where we know the markers (before treatment) and we know how strongly the cells responded to a variety of drugs.

Within the Gray dataset, we also needed to target one subclass of cell lines versus another, despite the fact that nearly all cell lines were actually cancerous. We thus divided the 45 cell lines into “aggressive” and “less aggressive” cell lines based upon whether they were basal-like in origin or luminal, respectively. Labeling of cell lines as basal-like and luminal had already been done in [12] (see Methods S1 for details). Grouping the cell lines in this way allowed us to formalize targeting one distinct population over another within that dataset.

### Analysis

To extract useful information from these large datasets we used the regularized Generalized Linear Model (GLM) framework implemented in Matlab. GLMs are a class of machine learning algorithms that extend least-squares regression to target variable distributions other than the normal distribution. GLMs essentially relate a linear combination of predictor variables (like gene expression) to the predicted variable (like probability of being a cancerous cell) by passing the linear combination through a special function, the inverse link function, 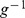. This link function is chosen to reflect the distribution of the predicted variable. The variable of interest, 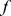, is related to the predictor variables, 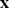 (e.g., a vector of genes' expression levels), and their weights, 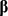 (or 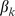
for each k^th^ gene), by:
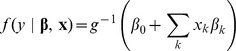



To prevent over-fitting, we implemented regularization. Regularization penalizes the algorithm for choosing a model that is too complicated – i.e., one with too many predictive features (genes). This forces it to choose only genes that contribute to goodness of fit. For the single-cell analysis in Part I, we used L2 (weaker) regularization because we expected that many of the 45 genes could be good predictors. For the drug treatment analysis in Part II, we used L1 (stronger) regularization because we expected that many of the ∼19,000 would not be good predictors. Regularization helped avoid fitting noise rather than signal, thus allowing us to capture the essential information in the data.

During estimation of the model parameters, regularization penalizes choice of 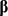 in proportion to its absolute value (L1) or its square (L2):
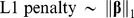


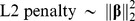



### Classification

In the first part of the study, we build a GLM to classify cells as cancer or healthy using single-cell PCR data. We use a GLM with the Bernoulli distribution. That is, we say that a cell either came from the healthy population (a value of 0) or from the cancerous population (a value of 1). Given the gene expression profiles for cells known to be healthy or cancerous, the GLM fitting returns a weight, 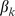, for each k^th^ gene according to how predictive that gene is. 
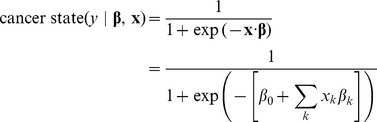



To ensure that our classifier generalized to data beyond training data, we used cross-validation (CV). We performed two levels of CV: 10-fold CV to determine the optimal value of the regularization penalty parameter (within the training set only), and 10-fold CV to test the predictions of the model. In this approach, we used 90% of the data to train the algorithm, and compared predictions made by that 90% to the actual values of the remaining naive 10%. We performed this ten times total, using a different 10% of the data for each round so that all of the data was eventually used as both training and validation data. This allowed us to show that our classifier worked with arbitrary data rather than just data it had already seen.

To quantify the accuracy of the GLM's predictions, we constructed Receiver Operating Characteristic (ROC) curves. An ROC curve characterizes the sensitivity and specificity of the classifier. Another way to quantify the overall performance of the prediction is to measure the area under the ROC curve. A perfect predictor algorithm would achieve an ROC integral value of 1, whereas a random predictor would achieve an ROC integral value of 0.5. ROC curves provided a straightforward way to interpret the accuracy of our classifier.

To measure the relative importance of measuring more markers versus more cells, we used smaller subsets of data to train the classifier. We did this by randomly choosing training data points (subsampling) for each of 100 iterations, for each training set size (2 cells through 95% of cells). For example, for a training set size of four data points, we chose a different set of four training points (two cancerous, two healthy) each of 100 times. Then for each set, we calculated the ROC integral (on strictly test data) as a function of the number of genes considered. We used a greater number of iterations for this analysis because training with a small number of points tends to be noisy (and generally undesirable for training classifiers). Thus, this approach allowed us to examine which factor contributes more to the performance of the algorithm: measuring more cells or more properties.

### Chemotherapeutic Response

In the second part of this study, we ask whether actual drugs act as classifiers. More specifically, we predict drug sensitivity of cell lines using gene expression data. To do this, we use the same GLM framework but now with a normal distribution because the original dataset defines sensitivity (-log(GI_50_)) as a continuous variable. As above, the GLM parameter for each gene reflects how well it predicts drug sensitivity. Modeling drug response as a GLM allowed us to ask whether drugs act like classifiers by using gene expression to determine their effects on cells.
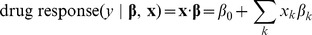



To test how well the predictions generalized to naïve data, we again used two levels of CV as in Part I (although here we use leave-one-out rather than 10-fold CV for measuring accuracy). We measured the performance of the algorithm with an *R^2^ value* rather than an ROC curve because drug sensitivity is a continuous variable. As above, we quantified the performance of the algorithm as a function of the number of genes considered. This allowed us to measure to what degree actual drugs act as classifiers.

To more clearly visualize drug sensitivity's dependence on gene expression, we produced drug sensitivity heatmaps. We fit a model using all of the data (rather than a subset), ranked genes based upon the parameters returned by the GLM, chose the best two, and then plotted drug sensitivity (“heat”) as a function of expression of each of the two genes. To smooth the heatmap, we used the Matlab function *griddata*.

### Treatment optimization

In the third part of this study we propose a method to optimize cancer treatment using the GLM framework. This consists of choosing two drugs that are more selective when combined than either drug alone. To do this, we build a classifier that discriminates between aggressive and less aggressive cancer (or ideally, healthy and cancer). We use only two features in this classifier: drug A's behavior (chemosensitivity, -log(GI_50_)), 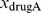, and that of another drug, 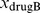. Thus, in terms of the drug behavior, 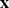, and their weights, 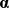 (which are distinct from 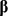 above):
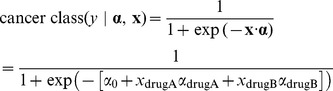



Where 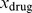 is given by how the drug's behavior (chemosensitivity) depends on gene expression, found as in Part II. 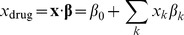



We then iterated through two-drug combinations to determine which one allowed the best discrimination (highest AUC) between aggressive and less aggressive cancer. Because overfitting is unlikely with two features (drug A behavior, and drug B behavior), we used a non-regularized GLM. As in Part I, we trained and tested the classifier with 100 rounds of subsampled data (90% train, 10% test) because there were a small number of data points. We estimated confidence intervals with bootstrapping. In practice, a full analysis should also correct for multiple comparisons as there are many possible two drug combinations. We have not performed such a correction, as this calculation was carried for demonstration purposes using a dataset that would not permit clinically relevant predictions.

## Supporting Information

Methods S1 **Includes details related to labeling of cell lines as basal-like or luminal as in Part III.**
(DOCX)
