# Supplementary material for: Conceptualizing Cancer Drugs as Classifiers
Source: PLoS One. 2014 Sep 23;9(9):e106444. doi: 10.1371/journal.pone.0106444 (PMC4172566; doi:10.1371/journal.pone.0106444)
Supplement: Methods S1 — Includes details related to labeling of cell lines as basal-like or luminal as in Part III. (DOCX) [file pone.0106444.s001.docx]

**Supplementary Methods**

*Data labeling for Part III: Treatment Optimization*

In Part III, we suggest one method to select drug combinations for screening that preferentially target cancer. Because healthy control tissues were not available in our dataset, we subdivided breast cancer tissues according to *subclass of cancer.* This does not represent a realistic treatment goal, and thus we do not propose that the results should generalize. The subdivision was grounded in physiological differences, but ultimately arbitrary and served to demonstrate the approach. Table S1 includes the names of the cell lines and their assigned labels.

**Table S1:**

| **Cell line** | **Cancer subtype** | **Label (aggressive = 1/less aggressive = 0)** |
| --- | --- | --- |
| 600MPE | Luminal (Lu) | 0 |
| AU565 | Lu | 0 |
| BT20 | Basal A (BaA) | 1 |
| BT474 | Lu | 0 |
| BT483 | Lu | 0 |
| BT549 | Basal B (BaB) | 1 |
| CAMA1 | Lu | 0 |
| HCC1143 | BaA | 1 |
| HCC1187 | BaA | 1 |
| HCC1395 | BaB | 1 |
| HCC1419 | Lu | 0 |
| HCC1428 | Lu | 0 |
| HCC1806 | BaA | 1 |
| HCC1937 | BaA | 1 |
| HCC1954 | BaA | 1 |
| HCC202 | Lu | 0 |
| HCC2185 | Lu | 0 |
| HCC3153 | BaA | 1 |
| HCC38 | BaB | 1 |
| HCC70 | BaA | 1 |
| LY2 | Lu | 0 |
| MCF7 | Lu | 0 |
| MDAMB134VI | Lu | 0 |
| MDAMB157 | BaB | 1 |
| MDAMB175VII | Lu | 0 |
| MDAMB231 | BaB | 1 |
| MDAMB361 | Lu | 0 |
| MDAMB415 | Lu | 0 |
| MDAMB436 | BaB | 1 |
| MDAMB453 | Lu | 0 |
| MDAMB468 | BaA | 1 |
| SKBR3 | Lu | 0 |
| SUM1315MO2 | BaB | 1 |
| SUM149PT | BaA | 1 |
| SUM159PT | BaB | 1 |
| SUM185PE | Lu | 0 |
| SUM225CWN | BaA | 1 |
| SUM44PE | Lu | 0 |
| SUM52PE | Lu | 0 |
| T47D | Lu | 0 |
| UACC812 | Lu | 0 |
| UACC893 | Lu | 0 |
| ZR751 | Lu | 0 |
| ZR7530 | Lu | 0 |
| ZR75B | Lu | 0 |
